# Cohesin complexes with a potential to link mammalian meiosis to cancer

**DOI:** 10.1186/2045-9769-2-4

**Published:** 2013-06-18

**Authors:** Alexander Strunnikov

**Affiliations:** Guangzhou Institutes of Biomedicine and Health, Molecular Epigenetics Laboratory, 190 Kai Yuan Avenue, Science Park, Guangzhou, 510530 China

**Keywords:** Spermatogenesis, Germline, Chromothripsis, CTCFL, BORIS, CTCF, SMC, Kleisin

## Abstract

Among multiple genes aberrantly activated in cancers, invariably, there is a group related to the capacity of cell to self-renewal. Some of these genes are related to the normal process of development, including the establishment of a germline. This group, a part of growing family of Cancer/Testis (CT) genes, now includes the meiosis specific subunits of cohesin complex. The first reports characterizing the *SMC1* and *RAD21* genes, encoding subunits of cohesin, were published 20 years ago; however the exact molecular mechanics of cohesin molecular machine in vivo remains rather obscure notwithstanding ample elegant experiments. The matters are complicated by the fact that the evolution of cohesin function, which is served by just two basic types of protein complexes in budding yeast, took an explosive turn in Metazoa. The recent characterization of a new set of genes encoding cohesin subunits specific for meiosis in vertebrates adds several levels of complexity to the task of structure-function analysis of specific cohesin pathways, even more so in relation to their aberrant functionality in cancers. These three proteins, SMC1β, RAD21L and STAG3 are likely involved in a specific function in the first meiotic prophase, genetic recombination, and segregation of homologues. However, at present, it is rather challenging to pinpoint the molecular role of these proteins, particularly in synaptonemal complex or centromere function, due to the multiplicity of different cohesins in meiosis. The roles of these proteins in cancer cell physiology, upon their aberrant activation in tumors, also remain to be elucidated. Nevertheless, as the existence of Cancer/Testis cohesin complexes in tumor cells appears to be all but certain, this brings a promise of a new target for cancer therapy and/or diagnostics.

## Introduction

Cohesin is a protein complex that is essential for cell proliferation in all eukaryotic cells. Cohesin is the key activity that establishes sister chromatid cohesion (SCC) and then holds sister chromatids until the anaphase. The separation of sister chromatids in mitotic cell division requires the inactivation of SCC function by either proteolytic cleavage or stripping cohesin molecules from chromatin. The original (“mitotic” or “somatic”) cohesin complex is postulated to have the shape of a “ring” that is potentially able to physically embrace two chromatids [1]. All cohesin complexes are composed of four essential subunits. The chromatid-embracing core of the ring-like structure is formed by two SMC proteins, SMC1 and SMC3, which belong to the family of SMC (Structural Maintenance of Chromosomes) ATP-binding proteins [2]. SMC1 and SMC3 heterodimerize by joining via their hinge domains [3]. The “locking” of the ring is achieved via binding of two ATP molecules at the ATP-binding domains of SMC1 and SMC3 [4]. Such a locking of the ring is facilitated by the third subunit that is known as RAD21/SCC1/MCD1 in a variety of systems [5–7]. This very subunit is the target of proteolytic cleavage by the separase/separin coincidental with anaphase initiation [8–10]. The forth core cohesin subunit is a HEAT repeat protein known as SCC3 in yeast [11], and represented in vertebrates by two paralogs, SA1/STAG1 and SA2/STAG2 [12, 13]. The functionality of mitotic/somatic cohesin is well studied with respect to its involvement in SCC at the centromeres. The molecular role of cohesin at the chromosomal arms is more obscure, however it has been directly linked to DNA repair [14] and, albeit less conclusively, to many instances of gene expression regulation [15–18]. It is believed that cohesin, in yet to be uncovered fashion, facilitates the function of the multipurpose transcriptional regulator CTCF [19], at a subset of thousands of CTCF sites in the genomes of Metazoa where cohesin and CTCF colocalize [20, 21]. However, as CTCF itself is a multifunctional protein, delineating the molecular pathway linking cohesin to gene expression regulation has proven rather difficult. Furthermore, while the differential functions of SA1/STAG1 and SA2/STAG2 are not well defined, the most recent data indicate that SA1/STAG1, but not SA2/STAG2, is somehow linked to cohesin colocalization with CTCF [22].

With the exception of still unclear mechanistic links to non-SCC mediated processes, such as transcription, the biggest splash since the characterization of cohesin in the 1990s was made with the discovery of the meiotic function of cohesin. Indeed, upon entering meiosis I, in the preparation for the reductional division, a meiosis-specific subunit REC8 de facto replaces RAD21/SCC1/MCD1 and makes mei-cohesin to behave rather differently with respect to timing of SCC1 release. Namely, REC8 protects SCC throughout the process of homologous recombination and then maintains the association of homologues after recombination is complete, up to the point when chromosomes are ready to segregate. At this point, REC8-mediated cohesion is released along the arms, but persists at the centromeres to ensure proper sister chromatid orientation in meiosis II [23–25]. In yeast, REC8 is known to have some functions in addition to SCC per se, including chromosomal restructuring leading to recombination: assembly of axial elements, pairing and synapsis of homologues. Yeast REC8 can easily take the place of SCC1/MCD1 in the SMC1/SMC3 complex in vitro [26], however in vivo studies in mice show that there is little, if any, exchange of REC8 once it becomes chromatin-bound [27]. In general, in metazoan systems, the rebuilding of cohesin for meiosis is much more complex, evidently due to the evolutionary emergence of the sophisticated germline development process. In addition to REC8 [28], there is a meiosis-specific paralog of SMC1α, SMC1β [29], as well as the meiosis-specific SA3/STAG3 subunit [30].

**Figure 1:**
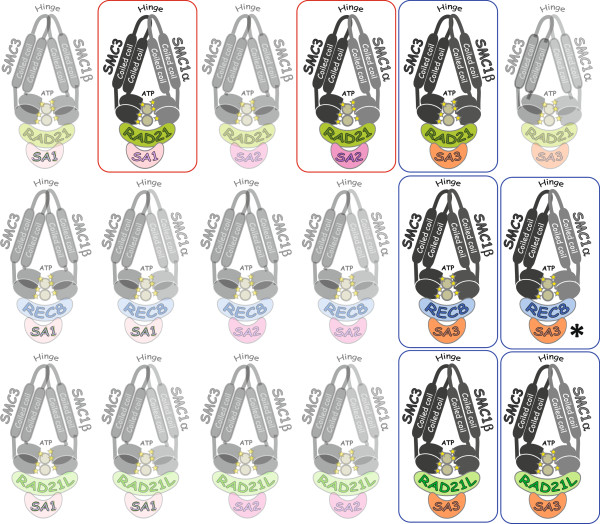
**Cohesin complexes based on all possible combinations of known cohesin subunits.** Meiosis-specific complexes are boxed in blue, and mitotic, which are also found in meiosis, are boxed in red. The complexes that were not validated biochemically are shown shaded. (*) Interaction of REC8 with SA/STAG subunits has not been studied exhaustively in mammalian systems.

Making the sense of the multitude of cohesin subunits is becoming objectively more and more difficult. Recently, three groups characterized the RAD21L (RAD21-like) protein, which is yet another meiosis-specific cohesin subunit [31–33]. RAD21L is expressed strictly in germline, i.e. in spermatocytes and oocytes. The presence of putative RAD21L complexes in the repertoire of meiotic cohesins significantly increases the level of potential complexity in the task of defining separate types of cohesin complexes in meiosis. A recent review on the subject estimates that there are 18 potential cohesin complexes (Figure 1) that could be present in cells, based on purely combinatorial considerations [34]. However, RAD21L cohesin complexes appear to come in only two forms in vivo: with SMC3 and STAG3 complexed with either SMC1α or SMC1β [31, 35, 36].

Rad21L appears at chromosomes in germline lineage in a fashion coordinated with REC8, at the pre-meiotic S phase. It resides on chromosomes from the establishment of SCC, through pairing of homologues and the establishment of the synapsis. More specifically, RAD21L appears to colocalize with axial elements upon meiosis initiation. Then, when homologues synapse, RAD21L stays associated with the synaptonemal complex all the way to the end of pachytene, when it disappears from the still persisting synaptonemal complex, according to [31]. However, there is some disagreement on the exact time of RAD21L disappearance [35], which is not possible to resolve based on purely cytological data. It is quite possible that REC8 is actually trailing RAD21L in chromosome loading in leptotene, making RAD21L the chief interface between cohesin and the initiation of synapsis. Upon cohabitation with REC8 the two proteins appear to enrich chromosomes in an alternating pattern [32], although high-resolution chromatin mapping data is required to confirm that that pattern is truly mutually exclusive.

The release of RAD21L from synaptonemal complex also coincides with the emergence of MLH1 foci at crossover sites. Thus, after the genetic recombination is finished, RAD21L leaves chromosomes, parting its ways with REC8, which stays bound at the axes of recombined chromosomes. As a result, RAD21L is apparently absent or has only marginal presence on chromosomes when they commit to segregation in meiosis I. This prompted a speculation that the RAD21L-containing cohesin represents the first case of cohesin complex which, at large, is actually uninvolved with SCC per se. Such a conclusion is indirectly supported by the observation that some of RAD21L loading onto chromosomes, apparently via the replacement of RAD21, in late pachytene may be replication independent [31, 32], i.e. happens after SCC establishment. However, if a direct exchange of RAD21 to RAD21L exists in situ, the situation becomes more complex to decipher. In any case, it would be premature to draw conclusions on any non-SCC function of RAD21L cohesin based solely on antibody staining data, without detailed molecular analysis and in the absence of mapping the distinct cohesin complexes in meiotic chromatin with high resolution.

## RAD21L cohesin complexes are involved in homologue pairing

The peculiar dynamics of chromosomal associations of RAD21L indicates that it may have a direct molecular role in homologue pairing. Alternatively, RAD21L and REC8 cohesin complexes may correspond to chromosomal regions marked by other epigenetic marks facilitating pairing. Such marks may also themselves predestine theses regions to either undergo recombination or to be refractory to it. Indeed, the pattern of alternation of Rad21L and REC8 on meiotic chromosomes creates an appearance of a “barcode” [31, 32]. The functionality of this “barcode” is awaiting the investigation. While pre-mitotic bar-coding remains a hypothesis, the direct role of RAD21L cohesin complex in homologue pairing is supported by cytological data. Namely, RAD21L, unlike other known cohesin subunits, does interact physically with SYCP1, a component of the synaptonemal complex. SYCP1 forms axial elements of the synaptonemal complex at the beginning of the pathway resulting in recognition and pairing of homologues [37].

While pairing of homologues is a feature of all eukaryotes with a meiotic cycle, RAD21L is present in vertebrates only. Unfortunately, however, little data on this protein is available in non-mammalian systems. In mammals, it is clear that at least some RAD21L cohesin complexes do colocalize as well as show signs of physical interaction with the proteins of axial elements, SYCP3 and SYCP2 [38, 39]. Mouse genetic data allow one to make the functional connection between cohesin subunits, axial elements’ structure, and genetic recombination itself. Single-gene knock-outs of *Smc1*β*, Rec8* and *Rad21L* show that spermatocytes arrest at the stage reminiscent of zygotene to pachytene transition, despite assembling axial elements and achieving a partial synapse between homologues [29, 33, 36, 40]. When both *Rec8* and *Rad21L* are deleted in mice, spermatogenic cells get arrested at a stage corresponding to leptotene, with chromosomes showing no pairing, apparently as a result of axial elements dysfunction. This stage approximately corresponds to the stage IV among the twelve morphological phases of seminiferous epithelium progression towards spermiogenesis. Spermatocytes with such a double knockout (dKO) stay viable up to that stage but later disappear, apparently as a result of arrest-induced apoptosis [36]. Such a dramatic meiotic defect of dKO has an inevitable consequence of infertility, i.e. all dKO males are sterile, visibly lacking any post meiotic cells in their seminiferous tubules, and with three-fold reduction in total testis size (at the age 6–8 week). While this phenotype is quite severe, it appears to be largely additive of similar defects in *Rec8*-deleted and *Rad21L*-deleted mice [33, 36, 40], indicating that REC8 cohesin complexes and RAD21L-containing complexes are specialized rather strictly.

In *Rec8*-*Rad21L* dKO spermatocytes, SYCP3 and SYCP2 fail to localize to chromosomes and the formation of axial elements is impaired. A punctate SYCP3 and/or SYCP2 staining reveals the accumulation of aggregates, which are devoid of SYCP1 [36]. However, chromosomes in mouse *Sycp1*-*Sycp3* dKO are still loaded with meiotic cohesin complexes, and the synapsis as well as recombination initiation both occur in such meiotic cells [41]. The sum of phenotypic analyses unequivocally indicates that either meiotic cohesin complexes are essential for the initiation of axial elements and the assembly of lateral elements or themselves are bona fide components of axial elements [36].

For understanding the underlying physiological abnormalities in dKO *Rad21L* and *Rec8* males mice it is important to consider that spermatocytes are not only defective in axial elements assembly, but are also unable to initiate a specific recombination pathway mediated by RAD51. This defect is signified by up to ten-fold reduction in RAD51 foci numbers observed in spermatocytes [36]. RAD51 normally assembles on bivalents within axial elements and lateral elements in leptotene and disappears later from all chromatin that was synapsed [42]. However, the number of DMC foci was unaffected in leptonema of the dKO spermatocytes, as well as the DMC1-dependent repair of DSB, which dKO generated (likely by SPO11) with normal frequency, was largely unaffected. Despite that, some repair detected by gamma-H2AX staining appeared to persist in chromosomes of arrested dKO spermatocytes [36]. This indicates either that the normal meiotic DMC pathway is partially perturbed, or that DSB were generated by a non-physiological mechanism. Taken together, this dKO spermatocyte phenotype indicates that while physiological DSBs priming recombination events are induced with normal frequency and kinetics, RAD21L and REC8 complexes are evidently involved in cooperative non-redundant pathways leading to axial elements formation followed by the assembly of the synaptonemal complex [36]. A direct involvement of meiotic cohesin complexes in genetic recombination may have substantial implications for cancer cells that express these proteins.

## Why another cohesin? Spermatogenesis vs oogenesis

KO of both *Rad21L* and *Rec8* in mice should have left the somatic cohesin fully intact in meiocyte lineage. Early binding of RAD21 to chromatin is indicative of its involvement in the establishment of SCC in the S phase preceding the meiosis [36]. Indeed, the proper establishment of SCC in the premeiotic S-phase seems unperturbed, by cytological criteria, while RAD21 remains chromosome-bound from leptonema to diplonema (or until pachytene in [32]) in mouse spermatocytes [36]. These observations seemingly suggest that meiotic cohesin complexes are not required for SCC, at least not as much as for their other functions, i.e. synaptonemal complex formation. On the other hand, in a specialized meiotic system in marsupials, STAG3 (most likely as a part of the incorporating cohesin complex) is involved in segregating sex chromosomes that undergo no recombination. This is achieved via assembly of a unique structure called dense plate [43]. These two polar considerations, i.e. the apparent un-involvement in SCC in mice and the participation in meiotic segregation that does not involve any recombination, both indicate that meiotic cohesins occupy a rather specialized niche in meiosis.

The exact degree of meiotic specialization of the three types of cohesin complexes built around RAD21, RAD21L or REC8 remains to be fully uncovered, however such a task may involve more than just analyzing the meiotic prophase. Indeed, their chromatin binding properties and turnover not just vary in the first prophase; they also behave differently through metaphases of both the first and the second meiotic divisions. The latter includes a substantial enrichment of RAD21L at centromeres at the metaphase II [33]. RAD21L is also the first cohesin subunit with a strong sex-dependent phenotype resulting from its inactivation. This somewhat challenges the hypothesis that the corresponding cohesin has a universal, essential and direct role in homologue pairing. In the context of such a hypothesis, it is difficult to explain why RAD21L appears to be distinctly distributed in male and female germlines. Namely, in the metaphase of meiosis I in spermatocytes, RAD21L is apparently left at the centromeres, while in oocytes it is not detectable at metaphase chromosomes [32]. This difference is not likely to be purely technical. Instead, it may either reflect a deep biological difference or stem from the distinctly differential dynamics of meioses in male and female germlines [44].

Genetic data also strongly indicate that RAD21L function is distinct in male and female germlines. RAD21L cohesin complex may be sex-restricted in its function, even though other meiotic cohesin subunits that were mutated did not show such a severe dichotomy. For example, SMC1β depletion leads to sterility in both males and females. However, the SMC1β-devoid mouse spermatocytes undergo an arrest in pachytene, while oocytes only suffer precocious dissolution of SCC during meiotic metaphase II [45]. Upon REC8 inactivation, both sexes in mice show sterility correlating with the impaired synapsis of homologues and the defective formation of chiasmata [40, 46]. At the same time, germ cells of male *Rad21L*−/− mice, as was mentioned, cannot achieve the complete synapsis of homologous chromosomes during the first meiotic prophase. As a result of this defect, and possibly others yet to be uncovered, spermatocytes run into zygotene arrest leading to the complete absence of spermatozoa. In contrast, female mice lacking RAD21L are fertile, but develop sterility at the age of 6 months. The molecular basis of this aging process is yet to be understood, as the original report seemingly excluded a probable defect in SCC as the leading cause, and the synapsed bivalents in KO females apparently have wild type amount of STAG3 loaded [33]. Furthermore, it appears that this difference between the sexes is not dependent on the differential regulation of meiotic checkpoints in males and females, particularly in relation to dictyate. Rather, this difference could be a reflection of the fact that RAD21L is not involved in homologue segregation in mouse oogenesis [33]. Thus, for RAD21L, we have evidence of both the sex-limited specialization and the redundancy with REC8. The latter is possibly reflected in evolutionary data that strongly suggest that only mammals need both proteins simultaneously, as RAD21L is not present in amphibians, while REC8 is absent in birds [35].

In the absence of data from non-mammal vertebrates it is not possible to judge whether such differences really represent the sex-specific functional properties of meiotic cohesins themselves, or result from a differential chromatin organization in sexes of placental animals. The latter, could be due to potentially differential roles of the global transcriptional regulator and chromatin-structuring factor CTCFL/BORIS in mammalian spermatocytes and oocytes [47, 48]. Indeed, in adult mouse testis, the CTCF paralog BORIS/CTCFL is expressed, likely transiently, in late spermatogonia and in the pre-leptotene germ cells [49]. An answer to the question whether BORIS/CTCFL facilitates, even if partially, RAD21L localization in male meiosis, considering that RAD21 notably co-localizes with CTCF in soma, will likely linger until one generates high resolution chromatin maps for meiotic cohesin complexes and compare them to the recent chromatin immunoprecipitation maps of CTCF and CTCFL in the germline [49].

## Cancer connection

The reference to CTCFL/BORIS brings about an important connection of some meiotic proteins to cancers. Indeed, germline-specific CTCFL/BORIS is aberrantly activated in many tumors and cancer cell lines [48, 50, 51], and its presence there was largely overlooked by the literature devoted to CTCF-cohesin colocalization in cancer cells of somatic origin. On the other hand, in the past several years it became evident that chromosome destabilization, as a result of mutation or epimutation, is likely one of the earliest steps in transforming a normal somatic cell into a cancer cell [52, 53]. Cohesin deregulation, in particular, may provide a rich potential source of such destabilizing changes, as the failure to resolve sister chromatid cohesion in mitosis universally results in chromosomal damage [54]. Indeed, both mutations of separase [55] and somatic cohesin subunits *SMC1L1* and *SMC3/CSPG6* themselves were correlated with tumors [56]. Tumor-borne mutations were also identified in the putative cohesin loader *SCC2/NIPBL* as well as in *STAG3*[56]. COSMIC database of cancer genomic data contains 15 examples of mutations in RAD21L, some of them reoccurring, 83 mutations in SMC1β, and 88 in STAG3. While REC8 activation in somatic cells has not been reported, its differential methylation was linked to poor cancer prognoses in gastrointestinal stromal tumor [57]. Similarly to RAD21L and REC8, the expression of SMC1β and STAG3 are known to be transcriptionally silenced in somatic cells, and this repression involves a set of diverse mechanisms, such as methylation of histone H3 on both lysine 9 and lysine 27 [58]. Despite such safeguards preventing the transcription of meiotic cohesin subunits in soma, RAD21L and other meiotic cohesin proteins are known to be aberrantly activated in cancers and thus fall into a special group of factors named Cancer/Testis genes, and Antigens [59–61]. RAD21L, SMC1β and STAG3 subunits have drastically distinct patterns of activation/overexpression in cancers, however. STAG3 activation is found practically in every dataset in Oncomine (September 2012 release), including all types of cancers, with some of datasets showing over 50% of samples overexpressing STAG3 over 2-fold. On the contrary, SMC1β is only expressed (over 2 fold increase over baseline) in 3.4% of samples in the same Oncomine release (public datasets), mostly in invasive breast carcinomas, and RAD21L is only found in 5 samples using the same cutoff. This indicates that the potential misregulation of somatic cohesin by STAG3 is a common event in cancers, while the expression of full meiotic cohesin, to include SMC1β and RAD21L, appears to be rare. It must be noted, however, that the baseline in gene expression experiments in somatic cancers may have been too high to detect a biologically meaningful activation of SMC1b and RAD21L. Furthermore, when the experimental approach is specifically targeted at CT genes, the outcome could be quite different. For example in a recently published dataset of only 33 cancer cell lines, six were shown to express RAD21L, with two co-expressing SMC1b with RAD21L [62]. Thus, as cohesin functions are intimately intertwined with chromosome segregation mechanisms, meiotic subunits could potentially serve as the source of epimutations leading to chromosome instability in cancers, especially considering how many “unnatural” complexes could be formed (Figure 1).

Among the biological and mechanistic questions about the nature of meiotic cohesins’ involvement in chromosome recombination and segregation in the norm, three especially stand out with respect to cancer biology. First, it is important to study whether somatic cohesin’s properties in vivo are altered upon STAG3 binding, as STAG3 activation and overexpression is a common event in multiple cancers. Second, it would be useful to determine whether the SMC1β-SMC3-RAD21L-STAG3 complex is actually able to participate in SCC. Third, it would be important to know whether RAD21L is cleavable by separase. If meiotic cohesin is able to establish SCC in principle, as the experimental results seemingly suggest, then a cancer cell where these proteins are activated may have a problem in resolving SCC in anaphase, recapitulating a well-characterized defect in SCC resolution [54]. This could only happen, if the meiosis-specific regulation of cohesin removal or a hypothetical RAD21L cleavage is not in place in mitosis in such cancer cells. It is plausible however, that mitotic prophase cohesin removal pathway mediated by PLK1 could remove RAD21L complexes in cancer cells similarly to the corresponding pathway engaged in meiosis I [32]. Nevertheless, the absence of cleavage of residual RAD21L complexes, particularly at centromeres where they can be targeted based on meiotic data [33], may pose a significant hurdle for chromosome segregation. Experimental investigation of these questions is feasible. While the ectopic expression of individual components of meiotic cohesin in cell lines does not appear to cause dramatic defects, neither correct stoichiometry was modeled nor detailed phenotypic analysis was performed in these studies with respect to chromosomes [35]. There were also no reports on the ectopic co-expression of all three meiotic subunits, SMC1β, STAG3 and RAD21L, in somatic cells.

Cancer cells may, however, lack the regulatory machinery to fully engage subunits of meiotic cohesin complexes, either ectopically expressed or aberrantly activated. It was recently reported that SMC3, STAG3, and REC8, as well as interacting factors SYCP2, SYCP3, HORMAD1, and HORMAD2, are specifically phosphorylated in the prophase I [63]. In case of SMC3, for example, the form phosphorylated at an ATM/ATR consensus mostly localizes to disjoined homologues’ regions, prior to synapsis. Furthermore, this regulation is probably tied into the DSB formation pathway, as SPO11 is required for it to occur [63]. Thus the experimental testing of the functionality of aberrantly activated meiotic cohesin is essential for understanding its molecular role in cancer cells.

Notwithstanding only limited information on the functionality of meiotic cohesin subunits in tumor cells, it is unlikely that their activation has an adaptive benefit for cell proliferation. On the contrary, such events are probably detrimental for chromosome integrity and segregation fidelity. Therefore, it is possible that the activation of meiotic cohesin genes in soma is involved in the earlier stages of tumorigenesis, thus providing a set of more universal targets for potential therapy compared to adaptive late-onset tumor mutations. Cancer/Testis genes were indeed reported as promising targets for the immunotherapy of cancer in model systems and in humans [64–66]. This strategy benefits from the tight repression of corresponding genes in soma, which enables one to diminish or eliminate potential side effects of treatments targeting these proteins. The activation of meiotic cohesin subunits may also provide an attractive target to find synthetic treatments targeting their combinations with other common mutations in cancers, especially ones in DNA damage response pathways.
